# Synergistic activity of cefepime/enmetazobactam with meropenem, piperacillin, piperacillin/tazobactam, cefiderocol and fosfomycin against *Klebsiella pneumoniae* carrying *bla*_KPC_ allelic variants

**DOI:** 10.1093/jac/dkag141

**Published:** 2026-04-11

**Authors:** Alice Crema, Ajwa Shafique, Gabriele Bianco, Paolo Gaibani

**Affiliations:** Department of Diagnostic and Public Health, University of Verona, Verona, Italy; Department of Diagnostic and Public Health, University of Verona, Verona, Italy; Department of Experimental Medicine, University of Salento, Lecce, Italy; Microbiology and Virology Unit, Azienda Ospedaliera Di Lecce, Lecce, Italy; Department of Diagnostic and Public Health, University of Verona, Verona, Italy; Microbiology and Virology Unit, Department of Pathology, Azienda Ospedaliera Universitaria Integrata Di Verona, Verona, Italy

Dear Editor,

The global dissemination of carbapenem-resistant Enterobacterales, particularly *Klebsiella pneumoniae* producing *Klebsiella pneumoniae* carbapenemase (KPC), represents a major therapeutic challenge in clinical microbiology and infectious diseases. KPC-producing strains are associated with high morbidity and mortality and have progressively reduced the effectiveness of many β-lactam antibiotics.^1^ The introduction of β-lactam/β-lactamase inhibitor combinations such as Ceftazidime/Avibactam significantly improved treatment options; however, increasing reports of resistance due to mutations in the *bla*KPC gene, particularly involving the Ω-loop region, have compromised their efficacy.^2^

Cefepime/enmetazobactam is a recently developed β-lactam/β-lactamase inhibitor combination composed of the fourth-generation cephalosporin and the penicillanic acid sulfone inhibitor Enmetazobactam. Enmetazobactam irreversibly inhibits class A serine β-lactamases by forming a stable acyl–enzyme complex, thereby protecting cefepime from hydrolysis.^3^ This inhibitor shows strong activity against extended-spectrum β-lactamases.^3^ Nevertheless, several studies have reported partial *in vitro* activity of cefepime/enmetazobactam against KPC-producing isolates, suggesting a potential therapeutic role in selected settings.^3–5^

In severe infections caused by multidrug-resistant Gram-negative bacteria, combination therapy is often employed to enhance antibacterial activity and limit resistance emergence. However, interactions between cefepime/enmetazobactam and other anti-Gram-negative agents remain poorly characterized.

Herein, we evaluate the *in vitro* interaction of cefepime/enmetazobactam with meropenem, piperacillin, piperacillin/tazobactam, cefiderocol, and fosfomycin against a collection of *K. pneumoniae* clinical isolates carrying different *bla*KPC allelic variants.

A total of 18 non-duplicate clinical isolates of KPC-producing *K. pneumoniae* recovered from tertiary-care hospitals in Italy between 2022 and 2024 were included. Isolates were selected to represent different *bla*KPC allelic variants. Molecular characterization by whole-genome sequencing was performed as previously described.^5^ MIC values were determined using MIC Test Strips (Liofilchem, Italy) according to EUCAST guidelines. Drug interactions were evaluated using the MIC Test Strip Synergy Application System and expressed as fractional inhibitory concentration index (FICI), while the magnitude of interaction was assessed using the susceptible breakpoint index (SBPI) as previously described.^6,7^

Baseline susceptibility testing revealed high resistance rates among the isolates. Overall, 94% of strains were resistant to piperacillin, 89% to piperacillin/tazobactam, 67% to meropenem, 72% to fosfomycin and 61% to cefiderocol. Resistance patterns differed slightly between wild-type and mutated KPC producers, with isolates carrying KPC variants generally showing lower carbapenem MICs but reduced susceptibility to newer β-lactam/β-lactamase inhibitor combinations (Table S1, available as Supplementary data at *JAC* Online).

Genomic analysis identified nine isolates carrying wild-type *bla*KPC genes (including *bla*KPC-2 and *bla*KPC-3) and nine isolates harbouring mutated variants (*bla*KPC-14, *bla*KPC-31, *bla*KPC-93, *bla*KPC-167 and *bla*KPC-205) (Table S2).

Cefepime/enmetazobactam demonstrated moderate *in vitro* activity, with approximately 70%–80% of isolates displaying MIC values within the susceptible range according to EUCAST criteria. Susceptibility was slightly higher among isolates harbouring mutated *bla*KPC variants compared with wild-type producers (Table S1).

Synergy testing showed that cefepime/enmetazobactam combinations produced predominantly indifferent interactions, ranging from 50% to 66.7% of isolates (Figure 1). At the same time, a major synergistic effect was observed for the combination of cefepime/enmetazobactam with cefiderocol, occurring in approximately 38.8% of isolates, followed by piperacillin (22.2%), meropenem (16.67%), piperacillin/tazobacta (22.2%) and fosfomycin (11.1%), and occasionally withother agents. No antagonistic interactions were observed for any antimicrobial combination tested.

**Figure 1.**
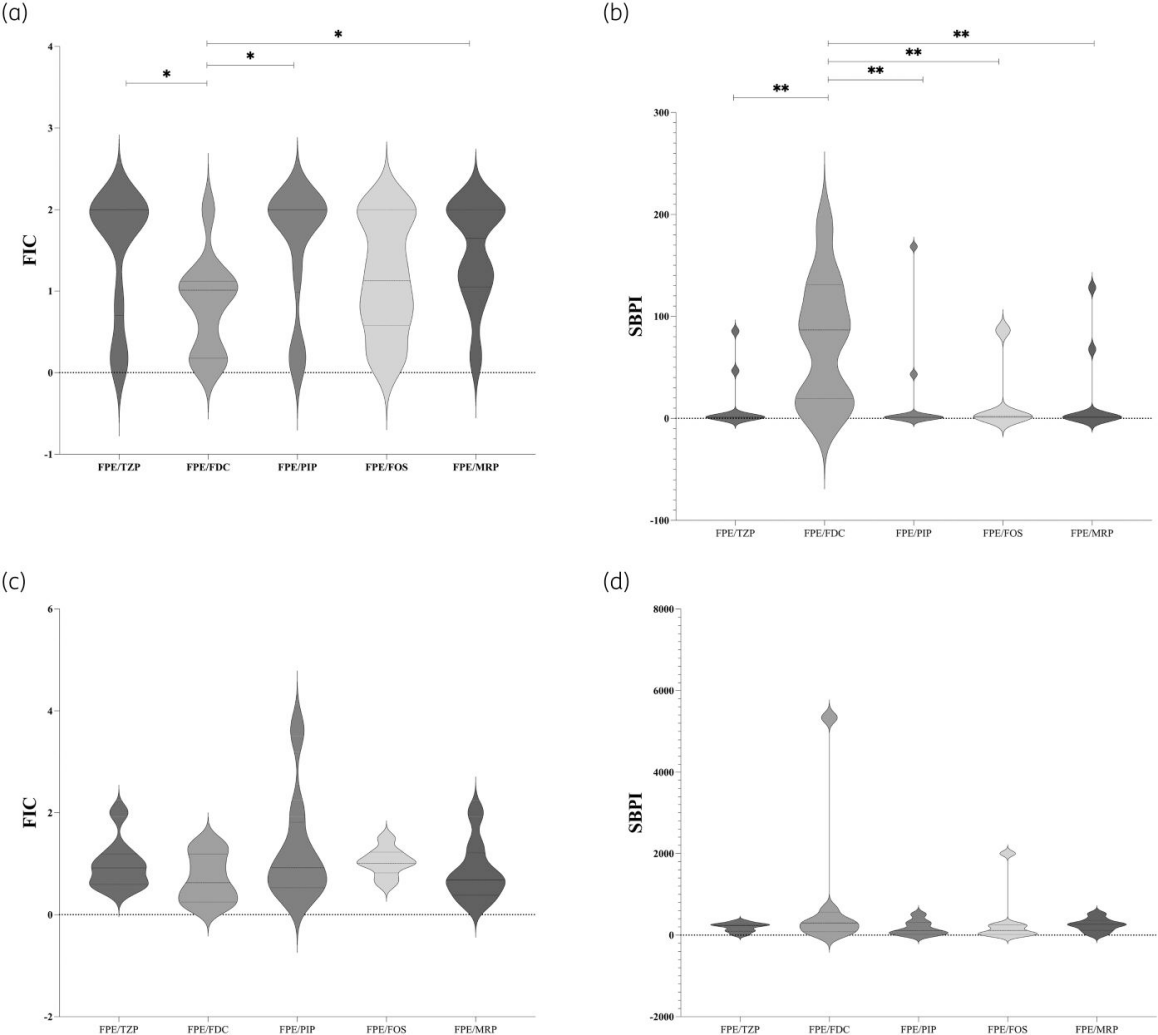
*In vitro* interaction of cefepime/enmetazobactam in combination with piperacillin, piperacillin/tazobactam, meropenem, cefiderocol or fosfomicyn against *Klebsiella pneumoniae* strains carrying wild-type and mutated *bla*_KPC_ gene. Panel A shows the FICI values of different combinations and Panel B shows the SBPI values.

Comparison of the FIC values between strains carrying wild-type and mutated *bla*_KPC_ gene showed that high synergistic activity was observed for cefepime/enmetazobactam with cefiderocol (median 1, IQR 1775–1.120) against strains harbouring wild-type alleles, while all the combinations displayed similar synergy effect against isolates carrying mutated KPC (Figure 1).

SBPI analysis revealed that the cefepime/enmetazobactam–cefiderocol combination (median 110, IQR 22.52–278.7) exhibited the highest overall antimicrobial potency, particularly against isolates carrying mutated *bla*_KPC_ genes (median 299.4 110, IQR 86–566). Similarly, against strains carrying wild-type *bla*_KPC_ genes, the major magnitude interaction was observed for the combination of cefepime/enmetazobactam with cefiderocol (median 86.62, IQR 19.49–131) (Figure 1).

In this study, cefepime/enmetazobactam showed moderate *in vitro* activity, consistent with previous surveillance studies reporting variable activity against KPC producers and susceptibility rates generally lower than those observed for ESBL-producing *Enterobacterales*.^3^

Synergy testing revealed that cefepime/enmetazobactam combinations were largely indifferent, suggesting that the addition of other β-lactams or non-β-lactam agents does not substantially enhance its activity *in vitro*. Nevertheless, the absence of antagonistic interactions indicates that cefepime/enmetazobactam can likely be safely combined with other antimicrobials when clinically indicated. Among the combinations tested, cefepime/enmetazobactam plus cefiderocol showed the most promising activity, with occasional synergistic interactions and higher SBPI values. This finding may reflect the complementary mechanisms of action of these agents, combining β-lactamase inhibition with the siderophore-mediated uptake of cefiderocol.^8^ Occasional synergy was also observed with fosfomycin, suggesting that this combination may represent an additional therapeutic option that deserves further investigation.

This study has some limitations, including the relatively small number of isolates and the *in vitro* design, which may not fully reflect clinical outcomes.

In conclusion, cefepime/enmetazobactam showed mainly indifferent but non-antagonistic interactions when combined with commonly used anti-Gram-negative agents against KPC-producing *K. pneumoniae*. Among the tested combinations, cefepime/enmetazobactam plus cefiderocol displayed the most promising *in vitro* activity, warranting further investigation in larger studies and *in vivo* infection models.

## Supplementary Material

dkag141_Supplementary_Data

## Data Availability

Data supporting this study are available upon reasonable request.

## Supplementary data

Tables S1 and S2 are available as Supplementary data at *JAC* Online.
