# Supplementary material for: Synergistic activity of cefepime/enmetazobactam with meropenem, piperacillin, piperacillin/tazobactam, cefiderocol and fosfomycin against Klebsiella pneumoniae carrying blaKPC allelic variants
Source: J Antimicrob Chemother. 2026 Apr 11;81(5):dkag141. doi: 10.1093/jac/dkag141 (PMC13070699; doi:10.1093/jac/dkag141)
Supplement: dkag141_Supplementary_Data [file dkag141_supplementary_data.docx]

**Table S1.** Antimicrobial susceptibility testing results of different antimicrobials tested against KPC-Kp carrying wild type *bla*_KPC_ and *bla*_KPC_ variant

| **Strain** | *bla*_KPC_ | **MIC (mg/L)** | | | | | |
| --- | --- | --- | --- | --- | --- | --- | --- |
|  |  | **PIP** | **TZP** | **MRP** | **FDC** | **FOS** | **FPZ** |
| 1 | MU | ≥256 | ≥256 | 1 | 4 | 16 | 0.32 |
| 2 | MU | 96 | 16 | 0.25 | 0. 016 | ≥256 | >64 |
| 3 | WT | ≥256 | ≥256 | ≥256 | 0.19 | ≥256 | >64 |
| 4 | WT | ≥256 | ≥256 | ≥256 | 0. 094 | 32 | >64 |
| 5 | MU | ≥256 | ≥256 | ≥256 | 4 | ≥256 | 8 |
| 6 | MU | ≥256 | ≥256 | 1.5 | 0.50 | ≥256 | 0.75 |
| 7 | MU | ≥256 | 48 | 1.5 | 0.75 | 24 | 2 |
| 8 | WT | ≥256 | ≥256 | 128 | 3 | 12 | >64 |
| 9 | WT | ≥256 | ≥256 | ≥256 | 24 | ≥256 | >64 |
| 10 | WT | ≥256 | ≥256 | ≥256 | 0. 094 | ≥256 | >64 |
| 11 | MU | ≥256 | 96 | 0.75 | 1 | 16 | 0.38 |
| 12 | MU | ≥256 | ≥256 | ≥256 | 8 | ≥256 | 1 |
| 13 | WT | ≥256 | ≥256 | 64 | 4 | ≥256 | >64 |
| 14 | WT | ≥256 | ≥256 | ≥256 | ≥256 | ≥256 | >64 |
| 15 | MU | ≥256 | ≥256 | ≥256 | 1 | ≥256 | 1.5 |
| 16 | MU | 8 | 4 | 0.25 | 0.75 | ≥256 | 0.75 |
| 17 | WT | ≥256 | ≥256 | ≥256 | 0.94 | ≥256 | >64 |
| 18 | WT | ≥256 | ≥256 | ≥256 | 32 | 16 | ≥64 |

**Abbreviations:** PIP, Piperacillin, TZP, Piperacillin/Tazobactam; MRP, Meropenem; FDC, Cefiderocol; FOS, Fosfomycin; FPZ, Cefepime/Enmetazobactam; WT, wild type; MU, mutated

**Table S2.** Genomic characteristics of KPC-Kp strains included in this study

| **Strain** | **ST** | **Serotype** | | **Antimicrobial resistance determinants** | | | | | | | | | | **Porins** | | **Plasmids** |
| --- | --- | --- | --- | --- | --- | --- | --- | --- | --- | --- | --- | --- | --- | --- | --- | --- |
|  |  |  | | **B-lactams** | | **Macrolides** | **Aminoglycosides** | **Quinolnones** | **Fosfomycin** | **Trimethoprim** | **Multidrug efflux** | **Sulfonamides** | **Tetracyclines** |  |  | **Inc Types** |
|  |  | **O** | **K** | **carbapenemase** | **B-lactamase** |  |  |  |  |  |  |  |  | **OmpK35** | **OmpK36** |  |
| 1 | 512 | O1/O2v2 | K107 | *blaKPC-31* | *blaTEM-1, blaSHV-11* |  | *aac(6’)-Ib* | *gyrA_S83I, parC_S80I* | *fosA* |  | *emrD, oqxA, oqxB* |  |  | truncated at aa 41 | ompK36_D135DGD | *ColRNAI, IncFIB(pQil), IncFII(K), IncN, IncX3* |
| 2 | 101 | O1/O2v2 | K17 | *blaKPC-167* | *blaTEM-1, blaSHV-1* | *msr(E), mph(E)* | *armA* | *gyrA-D87N, gyrA_S83Y, parC_S80I, qnrB1* | *fosA* | *drfA14* | *emrD, oqxA, oqxB20* |  | *tet(A)* | wt | ompK36_D135DGD | *Col156, ColRNAI, IncFIA(HI1), IncFII(K), IncR* |
| 3 | 307 | O1/O2v2 | K102 | *blaKPC-3* | *blaTEM-1, blaSHV-28* |  | *aph(3’)-Ia, aph(3’)-Ib, aph(6)-Ib* | *gyrA_S83I, parC_S80I, qnrSI* | *fosA* | *drfA14* | *emrD* | *sul2* |  | wt | ompK36_D135DD | *IncFIB(K), IncFIB(Mar), IncFIB(pQil), IncFII(K), IncHI1B* |
| 4 | 1519 | O1/O2v2 | K107 | *blaKPC-3* | *blaTEM-1, blaSHV-11* |  | *aac(6’)-Ib* | *gyrA_S83I, parC_S80I,* |  |  | *emrD, oqxA, oqxB* |  |  | truncated at aa 41 | ompK36_D135DGD | *Col(BS512), ColRNAI, IncFIB(K), IncFIB(pQil), IncFII(K),* |
| 5 | 101 | O1/O2v1 | K17 | *blaKPC-93* | *blaTEM-1, blaSHV-1* | *msr(E), mph(E)* | *armA* | *gyrA-D87N, gyrA_S83Y, parC_S80I,* | *fosA* |  | *emrD, oqxA, oqxB20* |  |  | wt | ompK36_D135DGD | *Col156, Col(MG828), ColRNAI, IncFIA(HI1), IncFIB(K), IncFII(K), IncR* |
| 6 | 1685 | O1/O2v1 | K17 | *blaKPC-14* | *blaTEM-1, blaSHV-1, blaOXA-1, blaCTX-M-15* | *msr(E), mph(E)* | *armA, aac(3)-Iie, aac(6’)-Ib-cr5* | *gyrA-D87N, gyrA_S83Y, parC_S80I* | *fosA* | *drfA14* | *emrD, oqxA, oqxB20* |  | *tet(A)* | wt | ompK36_T136TDT | *ColRNAI, IncFIA(HI1), IncFIB(K), IncFII(K), IncR* |
| 7 | 101 | O1/O2v1 | K17 | *blaKPC-31* | *blaSHV-1, blaCTX-M-15* | *msr(E),*  *mph(A), mph(E)* | *armA, aph(3’)-Ia, aph(3’’)-Ib, aph(6)-Id), aadA2* | *gyrA-D87N, gyrA_S83Y, parC_S80I, qnrSI* | *fosA* | *drfA12* | *emrD, oqxA, oqxB20* | *sul1/sul2* |  | wt | ompK36_T136TDT | *Col156, Col440II, ColRNAI, IncFIA(HI1), IncFIB(Mar), IncFII(K), IncR, IncHI1B* |
| 8 | 101 | O1/O2v1 | K17 | *blaKPC-2* | *blaSHV-1* | *msr(E), mph(E)* | *armA* | *gyrA-D87N, gyrA_S83Y, parC_S80I* | *fosA* |  | *emrD, oqxA, oqxB20* |  |  | wt | ompK36_T136TDT | *ColRNAI, IncFIA(HI1), IncFII(K), IncR* |
| 9 | 101 | O1/O2v1 | K17 | *blaKPC-2* | *blaTEM-1, blaSHV-1* | *msr(E), mph(E)* | *armA* | *gyrA-D87N, gyrA_S83Y, parC_S80I* | *fosA* |  | *emrD, oqxA, oqxB20* |  |  | wt | ompK36_T136TDT | *Col156, Col(MG828), ColRNAI, IncFIA(HI1), IncFIB(K), IncFII(K), IncR* |
| 10 | 1685 | O1/O2v1 | K17 | *blaKPC-2* | *blaTEM-206, blaSHV-1, blaOXA-1, blaCTX-M-15* | *msr(E), mph(E)* | *armA, aac(3)-Iie, aac(6’)-Ib-cr5* | *gyrA-D87N, gyrA_S83Y, parC_S80I, qnrB1* | *fosA* | *drfA14* | *emrD, oqxA, oqxB20* |  | *tet(A)* | wt | ompK36_T136TDT | *ColRNAI, IncFIA(HI1), IncFIB(K), IncFII(K), IncR* |
| 11 | 512 | O1/O2v2 | K107 | *blaKPC-31* | *blaTEM-1, blaSHV-11* | *mph(A)* | *aadA2, aac(6’)-Ib* | *gyrA_S83I, parC_S80I* | *fosA* | *drfA12* | *emrD, oqxA, oqxB* |  |  | truncated at aa 41 | ompK36_D135DGD | *ColRNAI, IncFIB(pQil), IncFII(K), IncN, IncX3* |
| 12 | 512 | O1/O2v2 | K107 | *blaKPC-167* | *blaTEM-1, blaSHV-11* | *mph(A)* | *aph(3’)-Ia, aadA2, aac(6’)-Ib* | *gyrA_S83I, parC_S80I* | *fosA* | *drfA12* | *emrD, oqxA, oqxB* | *sul1* |  | truncated at aa 41 | ompK36_D135DGD | *ColRNAI, IncFIB(K), IncFIB(pQil), IncFII(K)* |
| 13 | 101 | O1/O2v1 | K17 | *blaKPC-2* | *blaTEM-1, blaSHV-1* | *msr(E), mph(E)* | *armA* | *gyrA-D87N, gyrA_S83Y, parC_S80I* | *fosA* |  | *emrD, oqxA, oqxB20* |  |  | wt | ompK36_D135DGD | *ColRNAI, IncFIA(HI1), IncR* |
| 14 | 101 | O1/O2v1 | K17 | *blaKPC-3* | *blaTEM-1, blaSHV-1* | msr(E), mph(E) | *armA, aph(3’’)-Ib, aph(6)-Id, aadA1* | *gyrA-D87N, gyrA_S83Y, parC_S80I* | *fosA* | *drfA1* | *emrD, oqxA, oqxB20* | *sul1/sul2* | *tet(A)* | wt | ompK36_D135DGD | *Col156, Col440II, ColRNAI, IncFIA(HI1), IncFIB(AP001918), IncFII, IncFII(K), IncR, IncQ1* |
| 15 | 512 | O1/O2v2 | K107 | *blaKPC-205* | *blaTEM-1, blaSHV-11* |  | *aadA2* | *gyrA-D87N, gyrA_S83I* | *fosA* |  | *emrD, oqxA, oqxB* | *sul1* |  | truncated at aa 41 | ompK36_D135DGD | *IncFIB(K), IncFIB(pQil), IncFII(K)* |
| 16 | 101 | O1/O2v1 | K17 | *blaKPC-14* | *blaSHV-11* | *msr(E), mph(E)* | *armA* | *gyrA-D87N, gyrA_S83Y, parC_S80I* | *fosA* |  | *emrD, oqxA, oqxB20* |  |  | wt | ompK36_T136TDT | *Col156, Col(MG828), ColRNAI, IncFIA(HI1), IncR* |
| 17 | 512 | O1/O2v2 | K107 | *blaKPC-3* | *blaTEM-1, blaSHV-11, blaOXA-181, blaCMY-16* |  | *aph(3’)-Vib, aph(3’’)-Ib, aph(6)-Id, aac(6’)-Ib* | *gyrA_S83I, parC_S80I, qnrS* | *fosA* |  | *emrD, oqxA, oqxB* | *sul2* | *tet(A)* | truncated at aa 41 | ompK36_D135DGD | *ColKP3, IncA/C2, IncFIB(K), IncFII(K), IncX3* |
| 18 | 101 | O1/O2v1 | K17 | *blaKPC-2* | *blaTEM-1, blaSHV-1* | *msr(E), mph(E)* | *armA* | *gyrA-D87N, gyrA_S83Y, parC_S80I* | *fosA* |  | *emrD, oqxA, oqxB20* |  |  | wt | ompK36_T136TDT | *Col156, ColRNAI, IncFIA(HI1), IncFII(K), IncR* |
